# Antiproliferative and Pro-Oxidant Effect of Polyphenols in Aqueous Leaf Extract of *Passiflora alata* Curtis on Activated T Lymphocytes from Non-Obese Diabetic (NOD SHILT/J) Mice

**DOI:** 10.3390/antiox11081503

**Published:** 2022-07-30

**Authors:** Talita Cristina Colomeu, Daniella de Figueiredo, Priscila de Matos da Silva, Luís Gustavo Romani Fernandes, Ricardo de Lima Zollner

**Affiliations:** Laboratory of Translational Immunology, Department of Internal Medicine, School of Medical Sciences, University of Campinas—Rua Tessália Vieira de Camargo, 126, Cidade Universitária Zeferino Vaz, Campinas 13083-887, SP, Brazil; t108013@dac.unicamp.br (T.C.C.); daniellafig@gmail.com (D.d.F.); p1647712@dac.unicamp.br (P.d.M.d.S.); luisgrf@unicamp.br (L.G.R.F.)

**Keywords:** CD4^+^ and CD8^+^ lymphocytes, *Passiflora alata* Curtis, polyphenols, type 1 diabetes mellitus, NOD mice

## Abstract

The antioxidant, anti-inflammatory and antiproliferative properties of *Passiflora alata* Curtis are due to the presence of polyphenols in its composition. Our previous work showed that non-obese diabetic (NOD) mice undergoing treatment with aqueous leaf extract of *P. alata* present reduced insulitis in the pancreas, possibly due to its anti-inflammatory properties. However, depending on the concentration and their ability to interact with other molecules, these phenolic compounds may promote oxidation reactions in some cellular components, such as proteins and lipids, thus presenting a pro-oxidant effect. The present work aimed to evaluate the in vitro effects of aqueous leaf extract of *P. alata* and its polyphenols (vitexin, isoorientin, rutin and catechin) on lymphocyte proliferation and viability, the cell cycle and oxidative stress. Our results showed that T lymphocytes stimulated with concanavalin A mitogen (ConA) and in the presence of IC_50_ concentrations of *P. alata* extract and polyphenols undergo cell injury via inhibition of proliferation, with these effects being more pronounced concerning CD4+ T cells (*P. alata*, 3.54 ± 0.34%; isoorientin, 57.07 ± 6.4%; vitexin, 16.95 ± 1.11%; catechin, 37.9 ± 4.2% and rutin, 40.14 ± 4.5%), compared to the non-treated group (77.17 ± 6.29) (*p* < 0.0001 for all comparisons). This process includes late apoptosis/necrosis induction (*P. alata*, 77.5 ± 0.7%; vitexin, 83 ± 3.3%; isoorientin, 83.8 ± 1.4%; catechin, 83 ± 1.9% and rutin, 74.9 ± 3.2, while the control presented 53.6% ± 3.1 (*p* < 0.0001 for all comparisons)) and mitochondrial depolarization leading to cell-death induction. Furthermore, an in vitro model of a mixed culture of NOD mice T cells with a mouse pancreatic beta-cell line (MIN6) showed increased intracellular nitric oxide and lipid peroxidation in NOD T cells submitted to *P. alata* extract (46.41 ± 3.08) compared to the untreated control group (33.57 ± 1.99, *p* = 0.01315). These results suggest that aqueous leaf extract of *P. alata* and the polyphenols in these leaves represent a target for translational research showing the plant’s benefits for developing new drugs with immunomodulatory properties against inflammatory diseases such as diabetes mellitus.

## 1. Introduction

Polyphenols are secondary metabolites of plants [1] that can have anti-inflammatory, anticancer, vessel protective and anti-hepatotoxic properties, amongst other effects. Due to their anti-inflammatory properties, they have possible uses for treating different inflammatory diseases [2,3]. The effect of flavonoids, an important group of plant-derived polyphenols, has been studied in inflammatory and cancer cells [4]. The antiproliferative properties of polyphenols include inhibition cell proliferation and triggering cell cycle arrest [5], induction of apoptosis [6] and mitochondrial depolarization [7].

The genus Passiflora has about 500 known species, and *Passiflora alata* Curtis stands out due to the benefits of the phytotherapeutic compounds present in its leaves [8], which is mainly attributed to the high content of flavonoids in this plant structure. These compounds are associated with beneficial effects in controlling diseases initiated by oxidative stress by their antioxidant properties. The major flavonoid compounds of *P. alata* that present antioxidative activity include isoorientin, isovitexin, vitexin [9], rutin, catechin, epicatechin [10], apigenin and orientin [11].

However, the literature suggests that in addition to antioxidant effects, flavonoids also have a significant pro-oxidant impact under certain circumstances, such as certain concentrations and having the ability to interact with other molecules [12,13]. In their review, Lambert and Elias, 2010 [14] pointed out the pro-oxidant effect of green tea polyphenols in their capacity to increase reactive oxygen species generation, such as H_2_O_2_, in cell culture models. This could be attributed to the instability of these compounds in cell cultures, which can undergo autoxidative reactions resulting in the production of R.O.S. These authors also suggest that the pro-oxidant effects in animal models could be due to potential toxic effects generated by high dosages, which can generate pro-oxidant metabolites such as epigallocatechin quinone intermediates.

Reactive nitrogen species (R.N.S.) synthesis and its intermediate products are involved in pathophysiological mechanisms and complications in inflammatory diseases such as diabetes [15,16]. These molecules can initiate the oxidative stress process, which can induce D.N.A. damage and lipid peroxidation in cell membranes [15,16,17]. Although the causes of NO generation during adaptative immunity and inflammatory responses exerted by T lymphocytes are not fully understood, it is believed that NO is associated with tissue destruction through cytokine release induction and apoptotic stimulus activation [18,19]. On the other hand, under short-term exposure and low concentrations of R.N.S., immunoregulatory mechanisms such as inhibition of T cell proliferation [20,21] and platelet and neutrophil activation can occur [22], indicating a dual role of these molecules in inflammation.

A complex interaction among several types of cells, including T lymphocytes, occurs via biochemical events during the inflammatory process [23,24,25]. Among the proinflammatory cells that contribute to pancreatic insulitis, CD4^+^ and CD8^+^ T lymphocytes play a crucial role in insulin-producing beta cell injury, promoting hyperglycemia due to autoimmune type 1 diabetes mellitus (T1D) [26]. Non-obese diabetic mice (NOD mice) as one of the most fundamental experimental models of spontaneous T1D. Diabetes onset occurs from the 12th to the 24th week of life, with prevalence between 60–90% in females and 5–20% in males. In the 4th week of life, a few infiltrative mononuclear cells in the islets progressively invade the pancreatic islets [27], with the prevalence being CD4^+^ and CD8^+^ T cells. However, N.K. cells, B lymphocytes, macrophages and dendritic cells may also be found [28,29].

In our previous study, NOD mice treated with aqueous leaf extract of *P. alata* presented a decrease in proinflammatory infiltrative CD4^+^ and CD8^+^ lymphocytes (insulitis) and oxidative stress in the pancreatic islets [9], contributing to the survival of insulin-producing beta cells in the pancreas islets [10]. This study aimed to contribute knowledge of phenolic compounds’ in vivo anti-inflammatory effects via in vitro analyses. Aqueous leaf extract of *P. alata* and the polyphenols isoorientin, vitexin, catechin and rutin were analyzed, looking for their effects on cell proliferation, the cell cycle, apoptosis and apoptosis oxidative stress mechanisms in activated T lymphocytes from NOD mice mitogenic stimulus (Concanavalin A) or using the stressed insulinoma MIN6 as a source of β-cell antigen stimulus [30].

## 2. Materials and Methods

### 2.1. Lyophilized Passiflora alata Extract and Isolated Polyphenols

*P. alata* leaves (CNPq Patgen—010887/2014-8) were dried in a circulating air oven at 50 °C/48 h, ground and stored under refrigeration (8 °C). Subsequently, the aqueous leaf extract was filtrated in a 0.22 µm pore membrane, lyophilized and prepared to 0.1 g of leaves/mL water according to a previously described protocol [9]. The leaves lyophilization process was first diluted in phosphate-buffered saline (PBS), 0.1 M (pH 7.4) at 5 mg/mL concentration. The solutions of isoorientin, vitexin, catechin and rutin were prepared according to the manufacturer’s instructions (Sigma, St. Louis, MO, USA.), followed by dilution in PBS 0.1 M (pH 7.4). The specific dilution for the final concentration of *P. alata* extract and each polyphenol was then prepared in a culture medium for further cell treatment and filtered in a 0.22 µm pore membrane.

### 2.2. Mice

Female NOD ShiLt/J mice (*n* = 18) were obtained from the University of Campinas, Multidisciplinary Center for Biological Investigation on Laboratory Animal Science (C.E.M.I.B.), and maintained under temperature-, humidity- and light-controlled specific pathogen-free (SPF) facilities. The ShiLt/J non-obese diabetic (NOD) mice are an experimental animal model predisposed to spontaneous autoimmune diabetes and are widely used to study the immunopathology of type 1 diabetes, with close similarity to the human disease. Diabetes incidence in this lineage is 80–90% in females and 5–20% in males [31]. Thus, female NOD mice were grouped into six animals per experiment with triplicate cell analysis. NOD mice (30 weeks of life) were sacrificed by heart puncture under anesthesia (100–200 mg/kg ketamine hydrochloride and 5–16 mg/kg of xylazine hydrochloride (Dopalen, Vetbrands, Paulínia, Brazil).

### 2.3. Lymphocyte Proliferation Assay

The NOD mice spleens were aseptically removed, and the cell suspension was used to separate T lymphocytes. Briefly, spleen cells from three NOD mice were enriched for T lymphocytes through an adhesion protocol on nylon wool columns according to a previously described protocol [32] and stained with 1.25 µM carboxyfluorescein succinimidyl ester (C.F.S.E.) fluorescent probe (Thermo Fisher Scientific, Waltham MA, USA.) for 0.5 − 1 × 10^6^ cells. The lymphocytes were cultured in 96-well plates (Techno Plastic Products, Zellkultur, Trazadingen, Switzerland) at a concentration of 2.5 × 10^5^/well in RPMI 1640 culture medium (Gibco, Invitrogen Corporation, Grand Island, NY, USA) and supplemented with 10 U/mL penicillin (Gibco, Invitrogen Corporation, Grand Island, NY, USA), 5 μg/mL of Amphotericin B (Cristália L.T.D.A., Itapira, Brazil), 10 μg/mL streptomycin (Gibco, Invitrogen Corporation, Grand Island, NY, USA), 20 mM HEPES (Gibco, Invitrogen Corporation, Grand Island, NY, USA) and 10% fetal bovine serum (F.B.S.) (Thermo Fisher Scientific). T cells were submitted to a proliferation assay with the addition of 5 μg/mL of Concanavalin A (ConA) (Sigma Co, St Louis, MO, USA) in the cell cultures, as described previously [10]. To establish the IC_50_ inhibitory concentrations of *P. alata* extract and from polyphenols, different concentrations were established for *P. alata* (100, 300, 400 and 500 μg/mL) and polyphenols: isoorientin (Sigma) (25, 40, 80 and 100 μM), vitexin (Sigma) (50, 100, 200 and 300 μM), catechin (Sigma Co, St Louis, MO, USA) (10, 75, 100 and 500 μM) and rutin (Sigma) (100, 200, 300 and 500 μM). The treated lymphocytes and the control groups were incubated for 96 h at 37 °C in a humidified incubator in a 5% CO_2_ atmosphere (Lab Line Instruments Inc., Melrose Park, IL, USA). The effects of IC_50_ doses of *P. alata* extract and polyphenols were analyzed by CD4 and CD8 T cell subset proliferation assays. After washing procedures, the cells, previously stained with fluorochrome-conjugated monoclonal antibodies, were fixed with PBS 1% formaldehyde (Merck KGaA, Darmstadt, Germany) for acquisition in the flow cytometer as described below. The proliferating CFSE^low^ cells in the histograms presented in Figure S1 show the results as the frequency of proliferating cells.

### 2.4. Lymphocyte Mixed Cultures with Mouse Pancreatic Beta-Cell Line (MIN6) Cells

The mouse insulinoma cells (MIN-6) [33,34] at passages 22–30 were used as a source of pancreatic β cell antigens as described in previous work [30]. This cell lineage was kindly provided by Prof. Dr Antonio Carlos Boschero from the Department of Anatomy, Cell Biology, Physiology and Biophysics of the Biology Institute of Unicamp and maintained in a humidified incubator at a 5% CO_2_ atmosphere (Lab Line Instruments Inc, Dubuque, Iowa, USA) in a high-glucose D.M.E.M. culture medium (Gibco, Invitrogen Corporation, Grand Island, NY, USA) supplemented with 10% F.B.S., 200 U/mL of penicillin (Gibco), 200 μg/mL of streptomycin (Gibco, Invitrogen Corporation, Grand Island, NY, USA) and 5 μg/mL of amphotericin B (Cristália L.T.D.A., Itapira, Brazil). The medium was changed every 48 h, and the monolayers were passaged and used for experiments when 70% confluent.

MIN6 cells were transferred to 24-well culture plates at 8 × 10^4^/well and maintained for 18 h in D.M.E.N. at a final concentration of 100 mM glucose supplemented with 10% F.B.S. After that, 2.5 × 10^5^/well lymphocytes were obtained as described previously and were added to an RPMI 1640 medium at a final concentration of 11 mM glucose (stressing MIN6, the source of antigens to the culture medium) supplemented with 10% F.B.S. and 10 ng/mL of mouse recombinant (mr) IL-2 (PeproTech, Rocky Hill, NJ, USA) in each well. The mixed cultures were divided into the following groups: Untreated control group (C neg/control)—corresponds to a mixed culture of lymphocytes and MIN6 cells without any treatment; Treated group—corresponds to a mixed culture of lymphocytes and stressed MIN6 cells treated with the IC_50_ concentration of *P. alata* extract. After adding the lymphocytes and stimuli, the mixed cultures were incubated in a 5% CO2 atmosphere in a humidified incubator for 96 h. (30)

### 2.5. Cell Cycle Analysis

Enriched T lymphocyte cultures stimulated with ConA and treated with the IC_50_ dose of *P. alata* extract and polyphenols for 96 h were used to evaluate the frequency of cells in the G0/G1, S and G2/M phases of the cell cycle. To this end, a Guava^®^ Cell Cycle Reagent Kit (MerkMillipore, Burlington, MA, USA) was used to measure the D.N.A. content by propidium iodide (P.I.) staining in flow cytometry assays according to the manufacturer’s instructions. Briefly, cells were washed with 0.1 M PBS (pH 7.4), seeded at 2 × 10^5^ cells per well in 96-round-bottom-well plates (Techno Plastic Products, Zellkultur, Trazadingen, Switzerland), centrifuged at 400× *g* for 5 min, washed with PBS again, centrifuged and fixed with ice-cold 70% ethanol for 12 h at 4 °C. After this period, the cells were incubated with 200 μL of Guava^®^ Cell Cycle Reagent (MerkMillipore, Burlington, MA, USA) for 30 min at room temperature in the dark. The acquired samples were analyzed in flow cytometry, as detailed below. Figure S2 presents the representative histograms of cell cycle analysis showing P.I. fluorescence intensity and the markers that defined G0/G1, S and G2/M cell cycle phases.

### 2.6. Cell Death Assay

Enriched T lymphocyte cultures stimulated with ConA and treated with the IC_50_ dose of *P. alata* extract and polyphenols for 96 h were used to evaluate the frequency of cell death by apoptosis/necrosis. After culturing, the lymphocytes were washed with PBS 0.1 M (pH 7.4), centrifuged at 400× *g* for 5 min and stained with anti-CD4 and CD8 fluorochrome-conjugated monoclonal antibodies as described below. The cells were then washed with PBS 0.1 M (pH 7.4), centrifuged at 400× *g* for 5 min and stained with annexin-V and 7-Amino Actinomycin D (7-AAD) using an F.I.T.C. Annexin V apoptosis detection Kit with 7-AAD (Biolegend, San Diego, CA, USA) at the concentrations and with the procedures recommended by the manufacturer. Finally, 400 µL of Annexin V binding buffer was added to each tube, which was analyzed immediately via flow cytometry. We express the results as the percentages of viable, early apoptotic and late apoptotic/necrotic T cells. Figure S3 shows the representative dot plots demonstrating the quadrants that define the cell viability subsets.

### 2.7. Lipid Peroxidation Assays

Lipid peroxidation was evaluated in the MIN-6/T lymphocyte mixed cultures, treated or not treated with the *P. alata* IC_50_ dose, using Alexa Fluor™ 488 fluorescent probe linked to alkyne-modified linoleic acid. This compound suffers lipid peroxidation using copper-catalyzed click chemistry. To this was added, at the beginning of the culture period, the Click-iT^®^ Linoleamide Alkyne reagent (L.A.A.) (Thermo Fisher Scientific, Waltham MA, USA) at a concentration of 50 μM. After 96 h, the cells were washed with PBS and centrifuged for 5 min at 400× *g*. We used cell cumene hydroperoxide for positive control at 600 μM for 2 h at room temperature and protected from light. After washing with PBS, the cells were fixed, permeabilized and incubated with a reaction cocktail containing Alexa Fluor^®^ 488 azide (Thermo Fisher Scientific, Waltham MA, USA) for 30 min at room temperature and protected from light according to the manufacturer’s instructions. The washed samples were submitted for flow cytometry, and the results are expressed as the median of the fluorescence intensity of the Alexa Fluor™ 488 probe (Figure S4).

### 2.8. Nitrogen Reactive Species Detection

The viable CD4 and CD8 T cell subsets that present R.N.S. in the MIN-6/T lymphocyte mixed cultures, treated or not treated with *P. alata* IC_50_ dose, were assessed by flow cytometry using the fluorescent probe 4-amino-5-methylamino-2′, 7′-dichlorofluorescein diacetate (D.A.F.) (Thermo Fisher Scientific, Waltham MA, USA). After culturing, the cells were washed with PBS 0.1 M (pH 7.4), centrifuged at 400× *g* for 5 min and stained with anti-CD4 and CD8 fluorochrome-conjugated monoclonal antibodies as described below. After that, cells were labelled with 5 μM of D.A.F. for 40 min at 37 °C and protected from light. After washing procedures, the cells were fixed with PBS 1% formaldehyde. For positive control, mixed culture cells were incubated with sodium nitroprusside (SNP) (Sigma-Aldrich, St. Louis, MO, USA) at 0.2 μM for 30 min at 37 °C. The samples were analyzed via flow cytometry as described below. The results are expressed as the frequency of DAF^+^ cells in the CD4 and CD8 T cell subsets. Figure S5 presents the representative dot plots showing the quadrants used to define DAF^+^ cells in CD4 and CD8 T cell subsets.

### 2.9. Mitochondrial Depolarization Detection Assay

The mitochondrial membrane potential assay can be used to evaluate cell function and health; thus, cationic carbocyanine dye (JC-1) staining cells were used [35]. Thus, ConA-stimulated T lymphocytes exposed to IC_50_ doses of *P. alata* extract and polyphenols were washed with PBS 0.1 M (pH 7.4). After centrifuging at 300× *g* for 5 min at room temperature, the cells were labelled with 1 µg of JC-1 dye (Santa Cruz Biotechnology, Dallas, TX, USA), diluted in 100 µL of PBS and incubated at 37 °C for 30 min. The cells were then washed with PBS, centrifuged for 5 min at 300× *g* and analyzed immediately by flow cytometry. T cells were submitted to valinomycin (Cayman Chemical, Ann Arbor, MI, USA) as a positive assay control at 100 µM. The results are expressed as the percentage of depolarized (green fluorescent) and polarized (orange–red fluorescent) T lymphocyte mitochondrial membrane potential.

### 2.10. Oxigen Reactive Species Detection

Detection of the CD4 and CD8 T cell subsets that presented oxidative stress (ROS^+^), or mitochondrial superoxide activity (MitoSOX^+^) in live cells was by flow cytometry using the fluorescent probes CellROX^®^ green and MitoSOX™ red (Thermo Fisher Scientific, Waltham, MA, USA), respectively. ConA-stimulated T lymphocytes, after culturing with the IC_50_ doses of *P. alata* extract and polyphenols, were washed with PBS 0.1 M (pH 7.4), centrifuged for 5 min at 300× *g* and first stained with fluorochrome-conjugated antibodies as described below. The cells were then washed with PBS, centrifuged for 5 min at 300× *g* and stained with 5 µM CellROX^®^ green or MitoSOX™ red in 100 µL of PBS for 30 min at 36 °C in the dark. Immediately after staining, they were analyzed via flow cytometer. The results are expressed as the percentage of CD4 or CD8 subsets positive to R.O.S. or MitoSOX probes.

### 2.11. Flow Cytometry

To phenotype the CD4 and CD8 T cell subsets in the proliferation, cell death and reactive oxygen species-detection assays, the cell samples were stained with 0.25 μg/10^5^ cells with PerCP conjugated anti-mouse CD4 and CD8 monoclonal antibodies (Biolegend, San Diego, CA, USA) according to manufacturer’s recommendations. The acquisition of samples was performed on a Guava easyCyte flow cytometer (MerkMillipore, Burlington, MA, USA), and the data were analyzed using InCyte Software (MerkMillipore, Burlington, MA, USA) and the Guava cell cycle software package version 3.1.1 (Millipore) for cycle cells.

In the R.N.S. detection experiments, cells were labelled with anti-CD4 PerCP-Cy5.5 (Biolegend, San Diego, CA, USA) and anti-CD8 P.E. monoclonal antibodies (B.D. Biosciences, San Jose, CA, USA) at a concentration of 0.25 μg/2.5 × 10^5^ cells according to manufacturer’s recommendations. The viable population assessed by previous labelling with the Zombie NIR ™ viability probe (Biolegend, San Diego, CA, USA) was submitted to a FACSVerse™ flow cytometer (B.D. Biosciences, San Jose, CA, USA), followed by analysis on F.C.S. Express v6 software (De Novo Software, Glendale, CA, USA).

### 2.12. Statistical Analyses

Absolute IC_50_ was calculated using non-linear regression fit modelling in dose–response curves performed in the statistical package from GraphPad^®^ Prism v6 (La Jolla, CA, USA). We used one-way ANOVA Dunn’s multiple comparisons and two-way ANOVA Dunnett’s multiple comparisons for grouped analysis for statistical data analysis. The results are expressed by mean ± standard error and *p* < 0.05.

## 3. Results

### 3.1. IC_50_ Concentrations of P. alata Extract and Polyphenols

**Figure 1:**
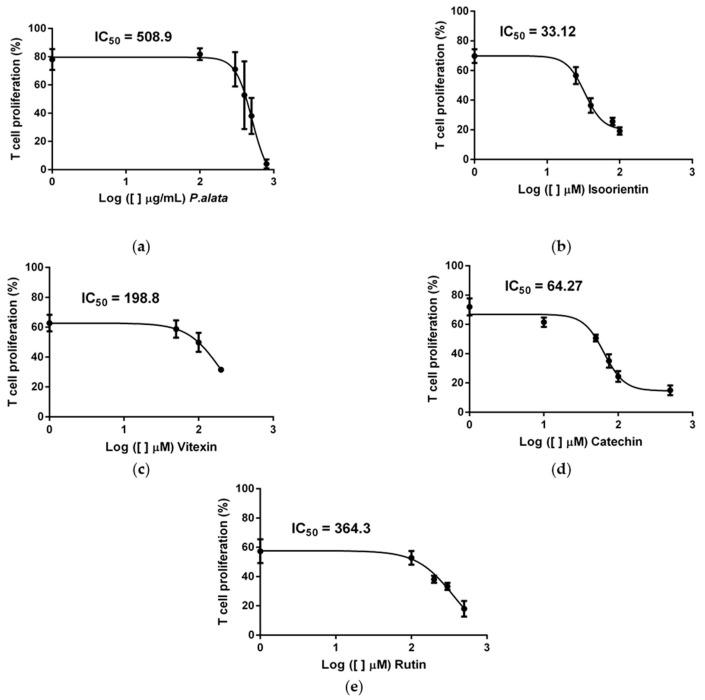
Effect of *P. alata* extract and polyphenols in Concanavalin A (ConA) proliferation-stimulated T lymphocytes. The dose–response curves of *P. alata* extract (**a**) and polyphenols: isoorientin (**b**), vitexin (**c**), catechin (**d**) and rutin (**e**) represent the IC_50_ of each respective compound calculated by non-linear regression fit modelling as described in the Material and Methods Section; *n* = 6 with triplicate cell assays.

According to our previous study [10], the extract of *P. alata* reduced the proliferation of activated T cells in a dose-dependent manner. Using dose–response curves in T cell proliferation assays, we show in Figure 1 that *P. alata* (a) and the polyphenols: isoorientin (b), vitexin (c), catechin (d) and rutin (e) present in *P. alata* leaves reduced ConA-activated T cell proliferation. To optimize the experimental procedures, we established for subsequent analyses the concentrations closest to the absolute IC_50_ for *P. alata* and polyphenols: *P. alata*, 500 µg/mL; isoorientin, 40 µM; vitexin, 200 µM; catechin, 75 µM; and rutin, 300 µM.

### 3.2. Cell Cycle Modulation of Lymphocytes at the IC_50_ of P. alata Aqueous Extract and Polyphenols

**Figure 2:**
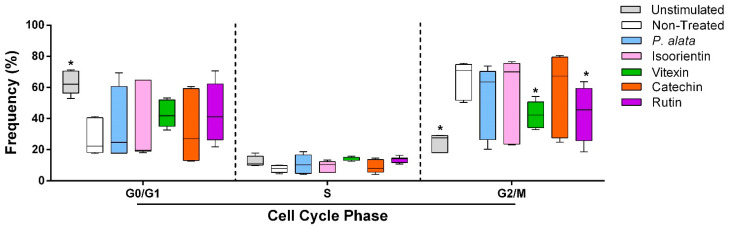
Effect of *P. alata* extract and polyphenols in the cell cycle of Concanavalin A (ConA)-stimulated T lymphocytes. The boxplots in panel represent the frequency of T cells in the G0/G1, S and G2/M phases of the cell cycle assay in the cell cultures stimulated with ConA and in the presence of IC_50_ concentrations of *P. alata* (blue) and polyphenols: isoorientin (pink), vitexin (green), catechin (orange) and rutin (purple). Gray boxplots represent T cells not stimulated with ConA, and white boxplots are the non-treated cells stimulated with ConA: * *p* < 0.05; *n* = 6 with triplicate cell assays.

The effects of *P. alata* and the polyphenols studied at their IC_50_ were investigated for cell cycle modulation by evaluating the frequency of lymphocytes in different cell cycle phases (G0/G1, S and G2/M). Cells treated with vitexin (42.3 ± 3%; *p* = 0.0261) and rutin (42.3 ± 6.3%; *p* = 0.0256) decreased the proportion of lymphocytes in G2/M compared to the non-treated group (65.8 ± 4.7%), with a tendency towards an increase in cells in the G0/G1 phase after these treatments (Figure 2).

### 3.3. Effect of Aqueous Leaf Extract of P. alata and Polyphenols on CD4^+^ and CD8^+^ T Lymphocyte Proliferation

**Figure 3:**
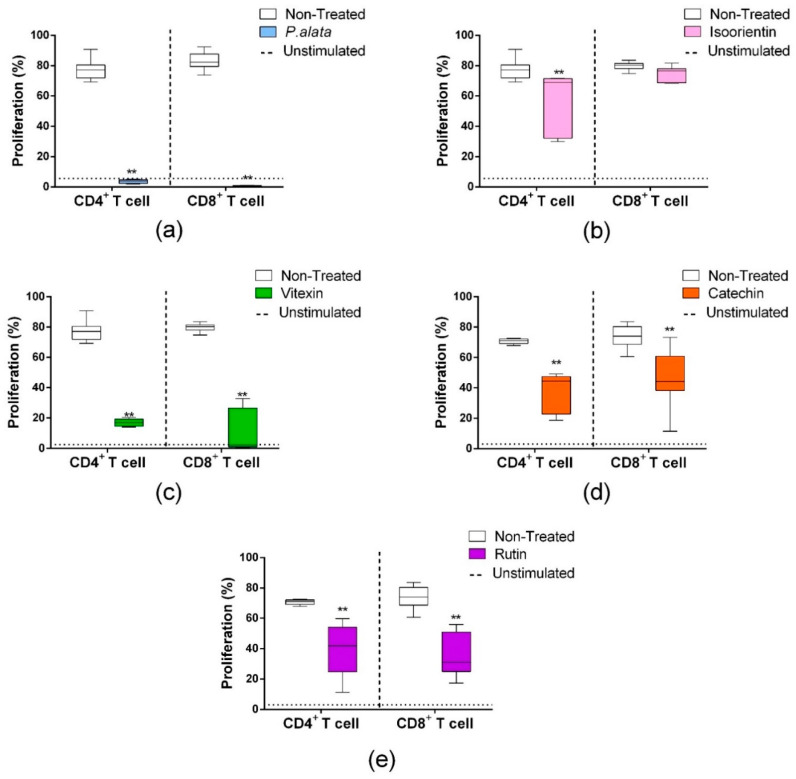
Effects of *P. alata* extract and polyphenols in the proliferation of CD4^+^ and CD8^+^ lymphocyte subsets. The boxplots represent the frequency of CD4^+^ (left) and CD8^+^ (right) proliferating T cells (CFSE^low^ population) treated (colored) or non-treated (white) with IC_50_ of *P. alata* extract (**a**) and polyphenols: isoorientin (**b**), vitexin (**c**), catechin (**d**) and rutin (**e**). Dotted lines represent the frequency of proliferating cells of non-stimulated lymphocytes: ** *p* < 0.0001, compared to the non-treated group; *n* = 6 with triplicate cell assays.

CD4^+^ T lymphocytes (Figure 3, left boxplots) treated with IC_50_ *P. alata* (a), isoorientin (b), vitexin (c), catechin (d) and rutin (e) showed reduced proliferations of 3.54 ± 0.34%, 57.07 ± 6.4%, 16.95 ± 1.11%, 37.9 ± 4.2% and 40.14 ± 4.5%, respectively, compared to the non-treated group (77.17 ± 6.29) (*p* < 0.0001 for all comparisons). Similar results in the CD8 subset (Figure 3, right boxplots) were found for the treatments with *P. alata* (0.56 ± 0.11%), vitexin (c) (11.11 ± 4.49%), catechin (d) (44.9 ± 5.1%) and rutin (e) (36.9 ± 3.6%) in comparison to the control group (83.01 ± 6.14) (*p* < 0.0001 for all comparisons). The only exception found was isoorientin (b), which presented no differences in cell proliferation compared to the non-treated group.

### 3.4. Effects of P. alata Aqueous Extract and Polyphenols on the Viability of CD4 and CD8 T Cells

**Figure 4:**
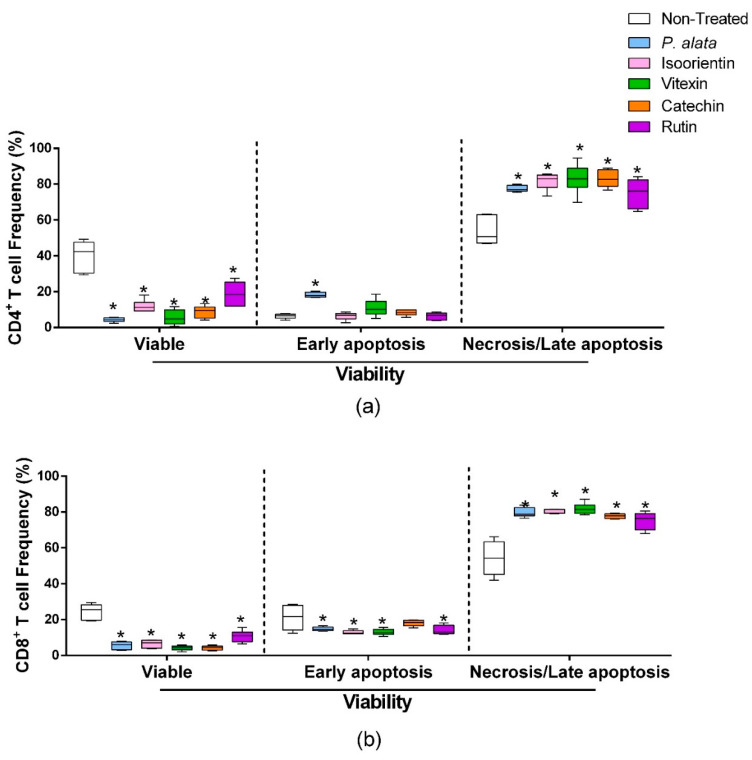
Effect of *P. alata* extract and polyphenols on viability and mitochondrial membrane potential. The boxplots represent the frequency of viable (left), early apoptosis (middle) and necrosis or late apoptosis (right) of CD4^+^ (**a**) and CD8^+^ (**b**) T cells stimulated with ConA and cultured in the presence of IC_50_ doses of *P. alata* extract, isoorientin, vitexin, catechin and rutin (colored boxplot). The cultures were treated with PBS only (non-treated, white boxplot) as a control: * *p* ≤ 0.05 compared to the non-treated group; *n* = 6 with triplicate cell assays.

ConA-activated CD4^+^ lymphocytes (Figure 4a) treated with IC_50_ of *P. alata*, isoorientin, vitexin, catechin, and rutin presented a significant decrease in cell viability (left boxplots) and increases in the proportion of lymphocytes in late apoptosis/necrosis (right boxplots). IC_50_ treatments increased the percentages of CD4^+^ cells in late apoptosis/necrosis, where values were: *P. alata*, 77.5 ± 0.7%; vitexin, 83 ± 3.3%; isoorientin, 83.8 ±1.4%; catechin, 83 ± 1.9%; and rutin, 74.9 ± 3.2%, while the control presented 53.6 ± 3.1 (*p* < 0.0001 for all comparisons). Concerning early apoptotic events, only the cultures treated with *P. alata* extract (18.25 ± 1.41%) present a difference (*p* < 0.0008) in comparison with the non-treated group (6.25 ± 1.32). *P. alata* extract and polyphenols had a similar effect on CD8^+^ T cells, decreasing the cell viability and increasing late apoptosis/necrosis. The percentages of late apoptotic/necrotic cells were: *P. alata*, 79.7 ± 1.1%; vitexin, 81 ± 1.2%; isoorientin, 77.5 ± 2.7%; catechin, 77 ± 0.5%; and rutin, 75.1 ± 1.9% and are statistically significant (*p* < 0.0001) in comparison with the non-treated group (54 ± 2.6%).

### 3.5. Effects of P. alata Extract on Membrane Lipid Peroxidation and Nitrogen Reactive Species (R.N.S.) Production in T Cells Co-Cultured with Pancreatic Beta-Cell Lineage (MIN-6)

**Figure 5:**
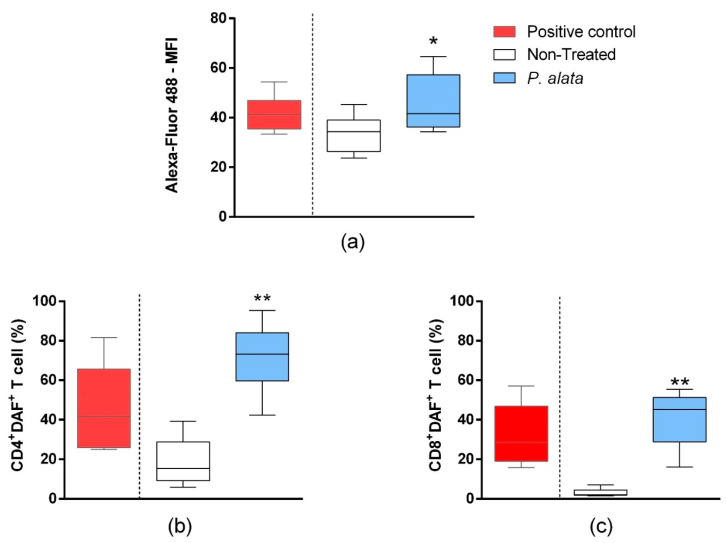
Effect of *P. alata* extracts on membrane lipid peroxidation and nitrogen reactive species production in T cells co-cultured with pancreatic beta-cell lineage (MIN-6). The boxplots (**a**) represent the median fluorescence intensity (M.F.I) of Alexa Fluor™ 488 fluorescent probe linked to alkyne-modified linoleic acid, which suffered lipid peroxidation using copper-catalyzed click chemistry (Click-iT Lipid peroxidation Imaging Kit—Thermo Scientific) in T cells treated (grey boxplots) or non-treated (white boxplots) with IC_50_ doses of *P. alata* extract. As a positive control of the assay, the culture was stimulated with the oxidizing agent cumene hydroperoxide at a concentration of 600 mM (black boxplots). The boxplots in panels (**b**,**c**) represent, respectively, the frequency (%) of CD4^+^ and CD8^+^ T cells presenting the 4-amino-5-methylamino-2′,7′-dichlorofluorescein diacetate probe (DAF^+^), which specifically detects nitric oxide inside the cells. Thus, as a positive assay control, the culture was submitted to nitric oxide donor sodium nitroprusside (SNP) at 0.2 mM (black): * *p* < 0.05; ** *p* < 0.0001; *n* = 6 with triplicate cell assays.

In the mixed culture of MIN6 cells and T cells, the total T lymphocyte population labelled by the Click-iT^®^ Linoleamide Alkyne (L.A.A.) kit using copper-catalyzed click chemistry linked to detect, after conjugation with Alexa fluor 488 fluorescent probes, alkyne-modified linoleic acid that suffered lipid peroxidation. In the IC_50_ *P. alata*-treated group, there was a significant increase (*p* = 0.01315) in the fluorescence intensity of Alexa fluor 488 (46.41 ± 3.08%), indicating an increase in lipid peroxidation in this group compared to the untreated control group (33.57 ± 1.99%) (Figure 5a). In agreement with this, there was a significant rise of R.N.S. (*p* < 0.0001) in CD4^+^ (71.77 ± 4.24%—DAF^+^ cells) and CD8^+^ (41.19 ± 4.49%—DAF^+^ cells) T cell subsets treated with an IC_50_ dose of *P. alata* extract in comparison with the non-treated group (19.10 ± 4.09% and 3.13 ± 0.53%, respectively) (Figure 5b,c).

### 3.6. Effect of P. alata Extract and Polyphenols in Mitochondrial Polarization of T Cells and Oxidative Stress in CD4^+^ and CD8^+^ T Cells Subsets

**Figure 6:**
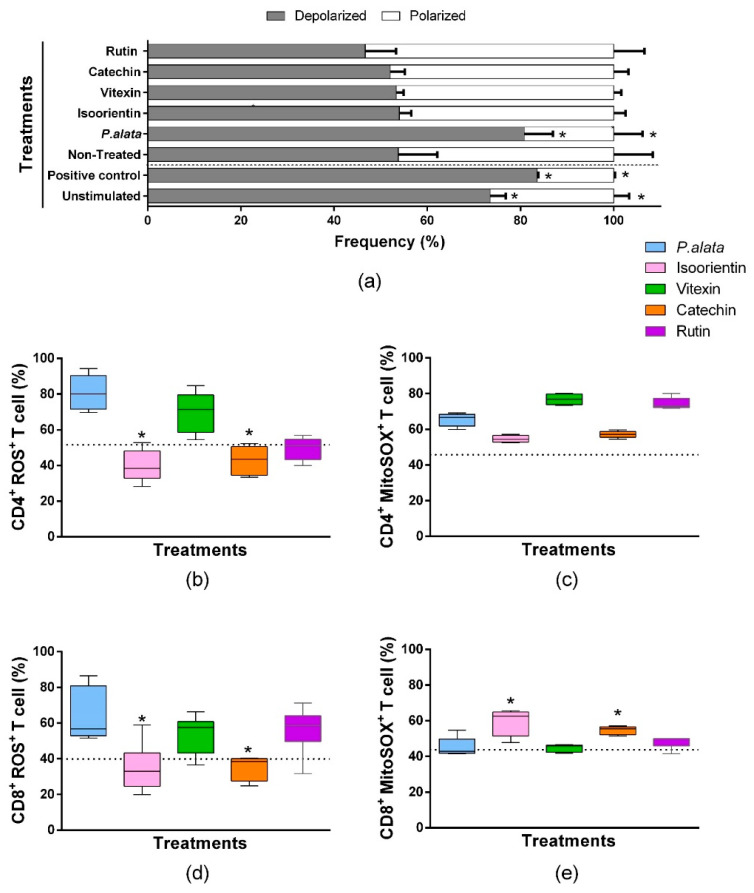
Oxidative stress analysis of CD4^+^ and CD8^+^ T lymphocyte subsets and mitochondrial depolarization in T cells stimulated with ConA and treated with IC_50_ doses of *P. alata* extract and polyphenols. The bars shown in panel graphic (**a**) represent the mean ± S.E.M. percentage of polarized (white bars) and depolarized (grey bars) mitochondrial membrane of cells treated with IC_50_ of *P. alata* extract, isoorientin, vitexin, catechin and rutin. Valinomycin at 100 µM was used as a positive control of mitochondrial depolarization. The non-treated cells were stimulated with 5 µg/mL of ConA, and T cells without proliferation stimulus or treatment (non-stimulated) are represented in the first pair of bars. The boxplots in panels (**b**,**d**) represent, respectively, the frequency (%) of CD4^+^ and CD8^+^ T cells presenting reactive oxygen species inside the cells generated exclusively by mitochondrial superoxide and detected with the MitoSOX™ fluorescent probe (MitoSOX^+^). The boxplots in panels (**c**,**e**) represent, respectively, the frequency (%) of CD4^+^ and CD8^+^ T cells presenting reactive oxygen species inside the cells detected with the CellROX™ fluorescent probe (ROS^+^). The cell cultures were stimulated with ConA and treated with IC_50_ doses of *P. alata* extract, isoorientin, vitexin, catechin and rutin (coloured boxplots). The results of non-treated cultures are represented as dotted lines in the graphs: * *p* < 0.05 compared to *P. alata*-treated group; group *n* = 6 with triplicate cell assays.

We assessed mitochondrial depolarization to evaluate cell death using a JC-1 fluorescent probe [35]. ConA-stimulated T lymphocytes treated with IC_50_ of *P. alata* presented mitochondrial depolarization in 80.7 ± 6.13% of the cells, significantly higher (*p* = 0.001) than the non-treated group (53.80 ± 8.35). The valinomycin-treated cultures (83.53 ± 0.275%) and non-stimulated cultures (73.49 ± 3.29%) show a significant increase (*p* = 0.0084) in the polarized population as well. However, the cell cultures treated with polyphenols presented no differences in mitochondrial depolarization values compared with the non-treated group. (Figure 6a).

A comparison of the effects of IC_50_ polyphenol treatments and *P. alata* extract treatment on oxygen reactive species (R.O.S.) production was assessed by the fluorescent probe CellROX^®^ green in flow cytometry assays. As demonstrated in Figure 6b, CD4^+^ lymphocytes treated with an IC_50_ dose of isoorientin and catechin presented lower values (40.04 ± 9.05% and 42.9 ± 8.31%, *p* = 0.0024 and *p* = 0.0032, respectively) in the frequency of ROS^+^ cells compared to *P. alata*-treated group (80.79 ± 9.93%). Similar results were observed for CD8^+^ lymphocytes compared to CD4^+^ lymphocytes (Figure 6d), where isoorientin (34.84 ± 13.54%; *p* = 0.0222) and catechin (35.09 ± 6.75%; *p* = 0.0281) showed a lower frequency of ROS^+^ cells in comparison with *P. alata*-treated group (63.97 ± 15.0%).

Mitochondrial superoxide is the principal reactive oxygen species that lead to oxidative stress and cell death. A Comparison of the effects among IC_50_ polyphenol treatments and *P. alata* extract treatment in superoxide (SOX) production was assessed by the fluorescent probe MitoSOX in flow cytometry assays. There were no differences in the frequency of CD4+ lymphocytes producing superoxide when comparing polyphenol-treated cultures with *P. alata*. However, *P. alata* (65.52 ± 3.61%; *p* = 0.041), vitexin (76.79 ± 2.90%; *p* < 0.001) and rutin (74.47 ± 3.26%; *p* = 0.0007) had higher superoxide production, which was significantly different from non-treated cells (45.67 ± 0.97%). Compared to the other treatments, *P. alata*, vitexin and rutin promoted higher concentrations of superoxide in CD4^+^ lymphocytes. CD8^+^ lymphocytes treated with isoorientin (59.38 ± 7.32%; *p* = 0.117) and catechin (54.74 ± 2.14%; *p* = 0.0246) showed higher expression of mitochondrial superoxide compared to treatment with *P. alata* (45.37 ± 5.19%).

## 4. Discussion

The hallmark of type 1 diabetes in NOD mice is the destruction of beta cells in the pancreatic islets by the immune system (mainly by T lymphocytes). This mechanism plays a crucial role in the insulitis process [36,37]. Thus, downregulation of T lymphocyte activation could be an excellent strategy to decrease inflammation, preserving beta-cell integrity [38,39].

In recent years, medicinal products from plants have been intensely investigated, mainly for their antioxidant and antiproliferative activities [40,41]. These plant properties may aid in treating inflammatory diseases such as diabetes mellitus and reducing oxidative stress, as demonstrated by the treatment of NOD mice with aqueous leaf extract from *P. alata* [9].

In the present study, the chosen IC_50_ inhibits 50% of lymphocyte proliferation. This process is the standard definition of a specific dose, as observed in some studies ([42] and [43]). Thus, we used this concentration to investigate: cell cycle, mitochondrial depolarization, oxygen and nitrogen reactive species, and mitochondrial superoxide enzyme activity.

Here, we show the dose-dependent effects of *P. alata* and polyphenols on T lymphocyte proliferation (Figure 1). Moreover, the efficiency of IC_50_ doses in inhibiting CD4^+^ and CD8^+^ lymphocyte subset proliferation (Figure 3) corroborates our previously published work, showing that treatment with aqueous leaf extract of *P. alata* inhibits lymphocyte proliferation [10]. The antiproliferative effect of *P. alata* leaves may be associated with effects due to the polyphenols present in the aqueous extract, such as isoorientin, vitexin, isovitexin, catechin, epicatechin and rutin, which may contribute to the anti-inflammatory effects in diabetic mice by decreasing inflammatory cells in the pancreatic islets [9,10].

In our previous work, the aqueous leaf extract of *P. alata* showed higher inhibition of T lymphocyte proliferation (BALB/c mice) than the treatments from another species of Passiflora [44]. The anti-inflammatory effects of *P. alata* leaves were also demonstrated by [45], showing reduced neutrophil and leukocyte migration after carrageenan-induced pleurisy in mice. Moreover, polyphenols found in *P. alata* leaves [9,10] have demonstrated in the literature anti-diabetic [46], antioxidant [47], anti-inflammatory [48] and antiproliferative effects [49].

Cell proliferation starts when cells enter the G1 phase, which allows the cell cycle to progress [50]. However, polyphenols can modulate the activity of some enzymes that induce T cell proliferation, such as tyrosine and serine-threonine protein kinases [51]. Our data show decreases in cells in G2/M after vitexin and rutin IC_50_ treatments and a trend towards an increased number of cells in G0/G1, suggesting modulation of these specific polyphenols on the cell cycle (Figure 2). Some polyphenols can affect the G1 phase, promoting arrest of the cell cycle. Epigallocatechin-3-gallate, a polyphenol belonging to the catechin family, induces G1 cell cycle arrest by downregulation of some cyclins, such as D and E, and the kinases CDK1, CDK2 and CDK4, and upregulation of p21 expression, a CDKI (cyclin-dependent kinase inhibitor) [52]. However, most studies considering the antiproliferative effects of polyphenols focus on cancer cells.

Studies demonstrate modulation of the cell cycle by the arrest of G2/M in cancer cells after treatment with vitexin [53] and rutin [54]. In addition, the IC_50_ of vitexin inhibits proliferation in cancer cell lineages such as mouse brain tumor (C6), human colon (HT29) and human cervical (HeLa) carcinomas [55]. The use of extract of *Clinacanthus nutans*, which has high concentrations of vitexin, suppressed the growth of A549 cells, a lung cancer line, demonstrating its chemopreventive action [56].

Our findings suggest that cell death mechanisms may affect the antiproliferative effect caused by *P. alata* and polyphenols in lymphocytes. The higher percentage of cells in late apoptosis/necrosis after treatment suggests that even with mitogen stimulus, the IC_50_ dose of *P. alata* and polyphenols can promote apoptosis in activated T lymphocytes (CD4^+^ and CD8^+^) (Figure 4). Indeed, both *P. alata* aqueous leaf extract and polyphenols induced a cell death mechanism in a dose-dependent manner (data not shown). Moreover, the antiproliferative effects of polyphenols in some cells may be due to apoptosis mediated by different pathways, such as cell membrane lipid peroxidation, mitochondrial depolarization, oxidative stress and production of mitochondrial superoxide.

Regarding lipid peroxidation in the lymphocyte cell membranes, our results showed in an in vitro cell co-culture model of the pancreatic beta-cell lineage (MIN-6) and NOD T lymphocytes that the IC_50_ dose of *P. alata* significantly increased this process in T cells (Figure 5a). Moreover, we found in both CD4^+^ and CD8^+^ lymphocyte subsets that treatment with the IC_50_ dose of *P. alata* induced a significant increase in nitric oxide in the active form in viable lymphocytes compared with control (Figure 5b,c). These data suggest a possible association between *P. alata* treatment and the induction of oxidative damage to CD4^+^ and CD8^+^ lymphocyte subsets, promoted by the increase in NO, and a consequent increase in lipid peroxidation. These facts present a plausible hypothesis since free radicals such as NO can inhibit or promote lipid peroxidation. As an inhibitor, NO acts by sequestering lipid radicals (peroxyl) and inhibiting enzymes initiating peroxidases. As an inducing agent, it promotes the formation of O.N.O.O., an important radical that initiates lipid peroxidation chain reactions [57].

Intrinsic apoptosis pathways may trigger mitochondrial depolarization due to the absence of growth factors and toxin-free radicals [58]. The polarized mitochondria are crucial for the transition as lymphocytes switch from quiescence to the proliferative state, and depolarization can occur due to the cytotoxic response, which leads to early apoptosis or necrosis [35] due to the membrane potential change (Δψ) [59].

Here we showed that after IC_50_ *P. alata* treatment there was a significant increase in mitochondrial depolarization (Figure 6a). Previous studies also demonstrated the effect of polyphenols in this pathway; Yuan, L. and coworkers [60] showed mitochondrial dysfunction induced in HepG2 cells (human liver cancer cell line) after isoorientin treatment. Moreover, studies with rutin also demonstrated apoptosis induction in proliferative cells [61]. At the same time, catechin obtained from *Ligaria cuneifolia* has antiproliferative properties, inducing apoptosis by mitochondrial depolarization and modulation of antiapoptotic proteins [6]. Mitochondrial depolarization can be activated by oxidants inducing R.O.S. (reactive oxygen species) overproduction and consequently initiating oxidative stress [62,63].

Concerning *P. alata* and polyphenols’ effect on R.O.S. generation in CD4 and CD8 lymphocytes, we observe differential behavior of the polyphenols when compared with *P. alata* extract. In both CD4 (Figure 6b) and CD8 (Figure 6d) subsets, isoorientin and catechin presented more reduced activation of R.O.S. production compared with *P. alata* treatment. Interestingly, in the CD8 subset (Figure 6e), we observed that these compounds induced higher mitochondrial superoxide activity when compared to the *P. alata*-treated group, which could indicate, beyond the differential properties of polyphenols, different action mechanisms of the same compound depending on the cell compartment. In this sense, other polyphenols in *P. alata* extract can contribute synergistically or by opposite effects, activating or inhibiting oxidative process, which could explain their dual role as pro- or antioxidant molecules depending on the circumstances and factors such as concentration and mechanism of interaction with other molecules [12,64].

Previous reports corroborating our findings have shown that treatment of mouse embryo fibroblast cells (M.E.F.) and mouse colon cancer cells (C-26) with the polyphenol quercetin causes necrotic death in cell cultures by a pro-oxidant effect due to generation of superoxide anion in the mitochondria [65]. In another study, epigallocatechin-3-gallate led to the production of mitochondrial R.O.S., preceded by the induction of both early–late apoptosis or a significant loss of mitochondrial membrane potential in human oral squamous cell carcinoma cells [66].

Therefore, our results suggest that *P. alata* extract and the phenolic compounds studied significantly affect lymphocytes. These effects include inhibiting proliferation, inducing pro-apoptotic pathways and modulating oxidative stress mechanisms such as R.O.S. and N.O.S. production, mitochondrial superoxide activity and cell membrane lipid peroxidation in CD4^+^ and CD8^+^ T cells. Previously, we showed that vitexin was the major component of aqueous leaf extract rather than isoorientin or isovitexin [9]. However, the effects observed by *P. alata* leaves may be related to several polyphenols exerting synergic/antagonist effects in regulatory pathways, as demonstrated by the differential behavior of the polyphenols investigated here.

Nevertheless, the chemical structure of most polyphenols contributes to their low bioavailability. Thus, because of poor absorption, they are retained in the intestine and gut barrier (a critical system for immunoregulation), affecting the intestinal microbiota [67]. We are finishing a manuscript concerning the effects of polyphenols on Tγδ intraepithelial lymphocytes and their properties in these cells. Therefore, polyphenols’ anti-inflammatory properties could mainly modulate the microbiota and the intraepithelial barrier cells [67,68].

Thus, these compounds must be studied more detail to understand and develop new drugs to treat chronic inflammatory diseases such as type 1 diabetes mellitus.

## 5. Conclusions

The aqueous leaf extract of *Passiflora alata* Curtis and its major polyphenols isoorientin, vitexin, catechin and rutin have antiproliferative properties that can contribute to the treatment of chronic inflammatory diseases. IC_50_ of *P. alata* extract and polyphenols, determined by lymphocyte proliferation analysis, can induce mitochondrial depolarization, thereby increasing oxidative stress by N.O.S., R.O.S. and mitochondrial superoxide, causing cell membrane lipid peroxidation and cell death in T lymphocytes. In addition, rutin and vitexin showed cell cycle modulation, demonstrating promising results for further investigating natural products that inhibit the upregulation of lymphocyte proliferation during the inflammatory process. These results suggest that aqueous leaf extract of *P. alata* and the polyphenols in these leaves could represent a target for translational research, showing the plant’s benefits for developing new drugs with immunomodulatory properties on inflammatory diseases such as diabetes mellitus.

## Data Availability

Data is contained within the article and supplementary material.

## Supplementary Materials

The following supporting information can be downloaded at: https://www.mdpi.com/article/10.3390/antiox11081503/s1. Datasheet S1: Original dataset sheets.
