# Supplementary material for: Antiproliferative and Pro-Oxidant Effect of Polyphenols in Aqueous Leaf Extract of Passiflora alata Curtis on Activated T Lymphocytes from Non-Obese Diabetic (NOD SHILT/J) Mice
Source: Antioxidants (Basel). 2022 Jul 30;11(8):1503. doi: 10.3390/antiox11081503 (PMC9405454; doi:10.3390/antiox11081503)
Supplement: Supplementary file 1 [file antioxidants-11-01503-s001.zip › antioxidants-1822209-supplementary.pdf]

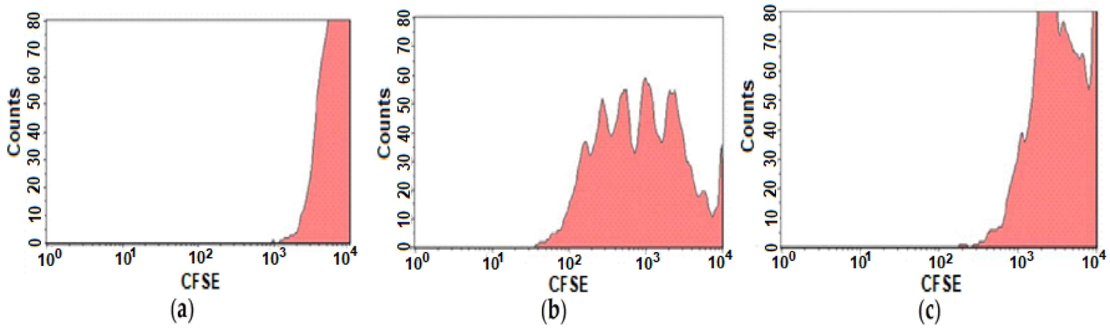

Figure S1. Representative image of the proliferation of CFSE-labeled lymphocytes analysis. Splenic T lymphocytes were treated cells with different concentrations of aqueous extract of *Passiflora alata* and stimulated cell proliferation with Concanavalin A (ConA). Panel (a) represents the histogram of untreated and unstimulated cultures presenting bright green fluorescent cells (right peak). Panel (b) shows the histogram of untreated and ConA stimulated cultures presenting di - viding cells showed by the fluorescent decay peaks. Panel (c) represents the histogram of *P.alata* extract - treated and ConA s timulated cultures giving a low frequency of dividing cells (right peak).

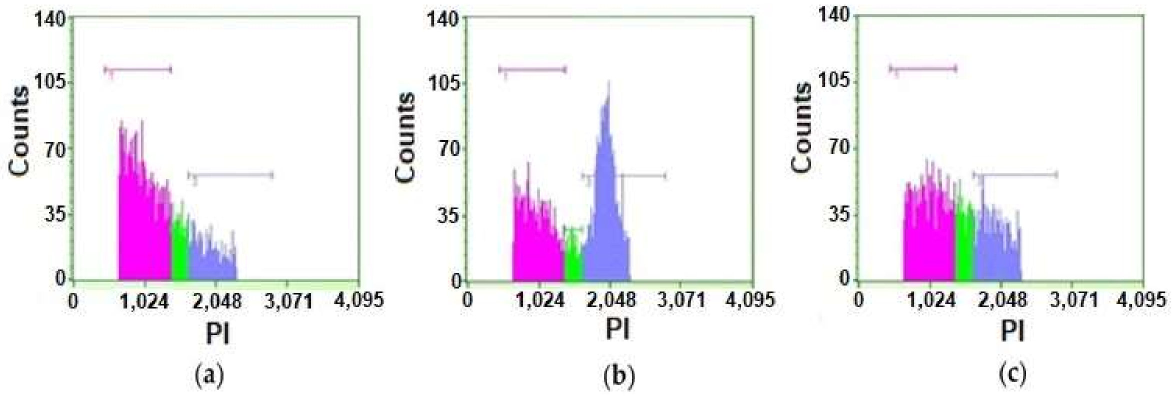

Figure S2. Representative image of cell cycle analysis by flow cytometry in T lymphocytes. The histograms show cells in G0/G1 (purple), S (green) and G2/M (blue). Panel (a) represents the histogram of untreated and unstimulated cultures, panel (b) represents the histogram of untreated and ConA stimulated cultures and panel (c) represents the histogram of *P.alata* extract-treated and ConA stimulated cultures.

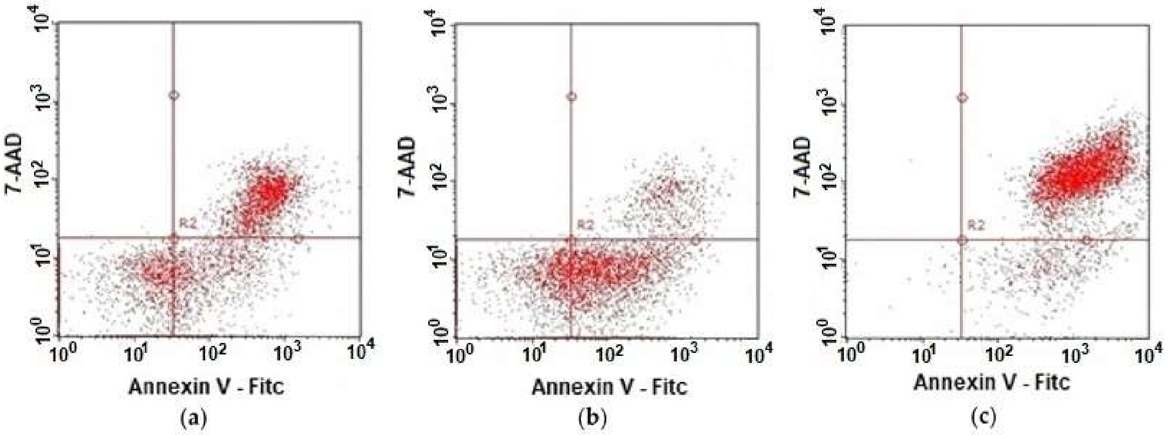

Figure S3. Representative image of cell viability assays by flow cytometry assays in T lymphocytes. Dot plots represent viable cells (Annexin V- 7-AAD-; low left quadrants), early apoptotic cells (Annexin V+7-AAD-; inferior right quadrant) and necrotic/apoptotic cells (Annexin V+7- AAD+; upper right quadrant). Panel (a) represents the dot plot of untreated and unstimulated cultures, panel (b) represents the histogram of untreated and ConA stimulated cultures and panel (c) rep-resents the histogram of *P.alata* extract-treated and ConA stimulated cultures.

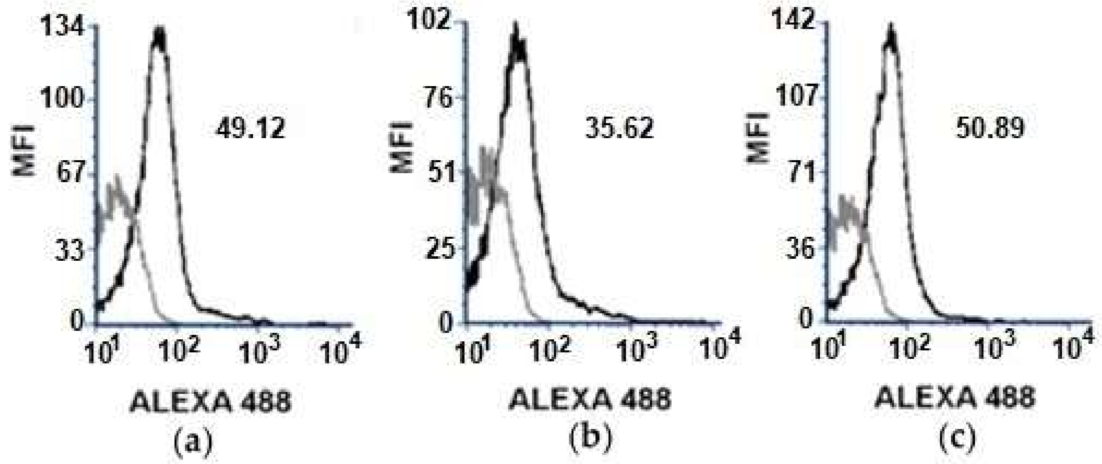

Figure S4. Representative histograms of the flow cytometry analysis of lipid peroxidation. The median fluorescence intensity (M.F.I.) of the Alexa Fluor™ 488 fluorescent probe indicates the alkyne-modified linoleic acid, which suffered lipid peroxidation using copper-catalyzed click chemistry (Click-iT Lipid peroxidation Imaging Kit–Thermo-Scientific) in T cells treated in mixed culture with MIN6 cells. The histogram in panel (a) represents the positive control of the assay (culture was stimulated with the oxidizing agent cumene hydroperoxide at the concentration of 600 mM); panel (b) represents non-treated cultures, and panel (c) represents the culture treated with IC50 doses of *P.alata* extract. The grey histogram in the graphs represents the unstained cells.

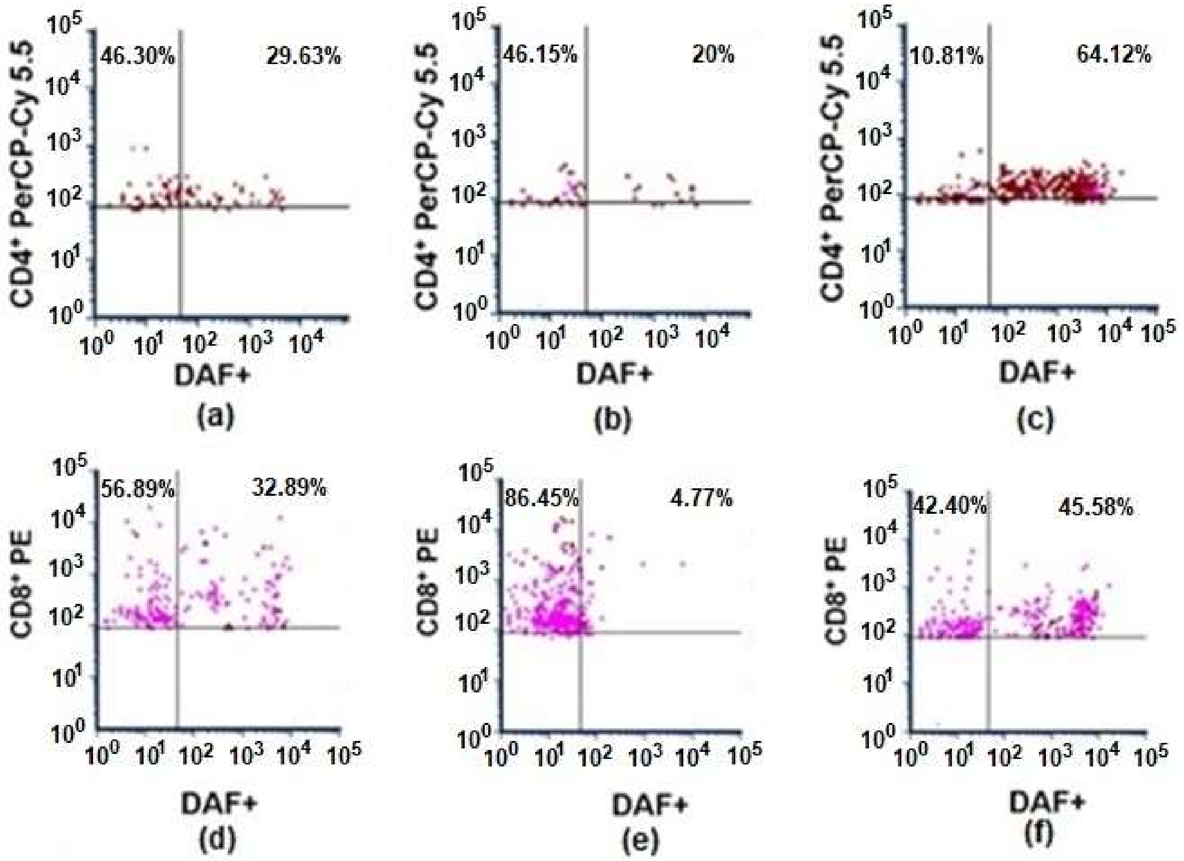

Figure S5. Flow cytometry analysis of nitric oxide detection in T cell subsets. Representative dot plots of the viable CD4+ (panels a to c) and CD8+ (panels d to f) T cells presenting the frequency of the 4-amino-5-methylamino-2',7′-dichlorofluorescein diacetate probe positive cells (DAF+ - upper right quadrants) in mixed culture with MIN6 cells. Panels (a) and (d) represent the positive control of the assay (culture was stimulated with mM the with the nitric oxide donor sodium nitroprusside (SNP) at the concentration of 0.2 mM); panels (b) and (e) represent non-treated cultures and panels (c) and (f) represents the cultures treated with IC50 doses of *P.alata* extract.

Datasheet S1

DATA FROM FIGURE 1

Log ([ ] µg/mL) *P.alata*	T cell proliferation (%)															
0	80.86	78.43	81.85	79.32	77.39	77.4	72.03	86.83	85.1	71.72	74.76	72.1	88.37	90.13	65.99	65.88
2	83.73	80.16	80.49	79.37	76.57	76.2	86.31	86.15	86.97							
2.477	61.56	51.65	52.54	76.57	59.34	73.94	82.62	85.07	74.83	81.54	84.56	69.27				
2.602	63.33	60.21	62.91	70.24	56.31	51.13	75.62	77.73	70.82	13.25	18.72	13.1				
2.698	22.31	19.16	13.25	42.24	41.46	38.49	43.18	46.47	40.41	49.3	53.44	47.11				
2.903	0.13	0.14	0.07	4.21	5.93	4.37	8.08	6.65	6.58							

Log ([ ] µM) Vitexin	T cell proliferation (%)											
0	68.37	71.81	66.14	57.55	66.15	61.17	59.59	57.03	56.75			
1.698	64.52	59.01	67.37	66.69	58.13	63.88	49.85	54.59	51.32	56.82	58.67	54.27
2	42.08	46.71	40.31	50.46	51	47.31	57.99	58.2	54.27			
2.301	32.32	30.34	30.24	34.46	33.82	33.55	29.2	30.85	30.51	31.66	30.06	

Log ([ ] µM) Isoorientin	T cell proliferation (%)								
0	64.4	73.11	73.24	69.05	75.23	73	67.25	62.79	
1.398	62.57	49.84	49.92	56.24	58.93	62.06			
1.602	40.62	38.74	43.95	33.01	31.95	39.64	32.28	31.26	
1.903	25.03	25.48	28.03	21.89	27.76				
2	22.03	22.26	18.99	20.48	19.44	20.84	14.8	16.83	17.5

Log ([ ] µg/mL) Rutin	T cell proliferation (%)											
0	55.47	68.91	71.58	45.93	55.58	50.6	57.53	54.8	55.46			
2	52.62	51.31	63.93	49.26	50.73	51.45	48.96	52.1	48.84	58.05	49.07	57.24
2.301	39.44	38.92	39.76	34.52								
2.477	33.75	36.29	31.7	34.91	35.77	29.52	30.77	33.34				
2.699	21.15	24.9	29	17.22	15.22	20.48	9.97	12.65	18.16	18.62	16.27	12.41

Log ([ ] µg/mL) Catechin	T cell proliferation (%)								
0	70.98	66.26	66.36	73.47	70.86	67.15	80.54	80.33	
1	60.47	65.04	61.68	66.69	58.69	62.21	60.63	56.79	
1.698	47.23	49.75	53.13	49.2	52.41	52.13			
1.875	36.36	33.55	36.95	37.41	39.5	26.47			
2	23.65	21.58	19.57	28.81	29.8	28.35	23.82	21.71	22.35
2.699	17.64	17.04	16.3	16.65	8.89	13.14			

DATA FROM FIGURE 2

	G0/G1							S							G2/M						
Unstimulated	71.3	70.5	66.3	62.1	61	52.9	56.4	10.4	11.3	12.4	10.2	9.8	17.9	15.8	18.2	18.1	21.2	27.7	29.1	29.2	27.8
Untreated	40.3	41.4	20.7	23.8	17.7	18.5		7.5	8.2	4.5	5.8	9.8	10.1		52.2	50.4	74.7	70.4	75.5	71.4	
P. alata	69.4	33.4	60.7	24.8	22.1	17.8	17.8	10.3	9.7	12.8	4.8	4.1	18.7	16.7	20.3	56.9	26.5	70.4	73.8	63.5	65.5
Isoorientin	64.7	60.8	64.8	19.1	18.1	19.6	19.6	12.3	13.4	11.6	5.4	5.3	10.3	10.4	23.1	25.9	23.6	75.5	76.6	70.1	70
Vitexin	52	47.8	53.3	40.1	41.9	35	32.7	15.1	15.1	12.5	15	15.8	14.2	13.2	32.9	37.1	34.2	44.9	42.3	50.8	54.1
Catechin	57	60.5	59.3	16.4	27	13.1	12.7	13.7	14.6	13.1	4.1	5.6	6.3	8	29.4	24.9	27.6	79.5	67.4	80.5	79.3
Rutin	70.6	48.5	62.3	37.7	41.2	26.4	21.9	10.8	16.4	11.8	14.6	13.2	14.2	14.4	18.6	35.1	25.9	47.6	45.6	59.4	63.7

DATA FROM FIGURE 3

Non-Treated

CD4$^+$ T cell	74.51	69.79	76.81	90.72	78.2	74.54	78.12	77.89	71.17	72.09	70.07	69.22	72.62	77.52	79.31	83.87	84.69	87.99
CD8$^+$ T cell	87.6	87.57	73.68	73.96	81.15	86.4	83.69	91.57	91.64	92.4	83.51	79.19	80.09	80.36	74.71	80.78		

Treated (*P.alata*)

CD4$^+$ T cell	4.69	5.02	3.75	3.66	2.25	1.89	4.69	5.02	3.75	3.66	2.25	1.89
CD8$^+$ T cell	0.64	0	0.32	0.98	0.4	1.07	0.64	0	0.32	0.98	0.4	1.07

Treated (Isoorientin)

CD4$^+$ T cell	33.25	30.92	29.92	71.66	68.84	69.84	66.29	71.35	71.54
CD8$^+$ T cell	76.67	76.56	68.39	76.38	81.7	68.72			

Treated (Vitexin)

CD4$^+$ T cell	20.47	16.87	15.19	14.1	18.15				
CD8$^+$ T cell	25.81	27.42	32.78	1.22	1.34	0.72	7.91	0.54	2.25

Treated (Catechin)

CD4$^+$ T cell	44.37	44.1	45.13	49.17	47.17	47.53	21.34	18.66	24.11					
CD8$^+$ T cell	44.71	39.35	38.28	44.21	41.8	38.21	11.36	12.09	17.29	72.5	73.17	64.47	58.96	56.18

Treated (Rutin)

CD4$^+$ T cell	51.69	59.71	48.59	53.27	54.9	57.46	19.93	14.78	11.17	41.91	40.89	37.91	29.69	
CD8$^+$ T cell	53.7	54.76	55.85	50	46.45	47.55	23.53	17.28	24.93	27.5	30.51	29.16	31.46	25

DATA FROM FIGURE 4

CD4+ T cell Viability

	Non-Treated						*P. alata*						Isoorientin					
Viable	47.09	49.17	45.25	39.32	30.59	29.41	3.76	4.39	4.13	5.74	5.36	2.38	12.63	10.65	11.69	18.04	9.09	9.39
Early apoptosis	5.63	4.02	6.81	7.34	6.06	7.64	16.93	20.18	19.65	17.88	16.78	17.69	7.58	6.53	2.6	8.63	7.44	5.63
Necrosis/Late apoptosis	47.28	46.81	48.25	53.34	63.35	62.94	79.31	75.43	76.23	76.38	77.86	79.93	79.8	82.82	85.71	73.34	83.48	84.98

	Vitexin						Catechin						Rutin					
Viable	0.45	9.43	5.78	2.48	11.63	3.97	8.21	5.56	13.28	4.24	10.83	10.73	18.47	11.92	11.76	18.35	24.69	27.45
Early apoptosis	5	9.43	10.98	8.42	18.6	13.25	7.5	5.56	9.96	8.05	9.75	8.29	4.03	3.97	6.27	7.15	8.68	7.79
Necrosis/Late apoptosis	94.55	81.13	83.24	87.11	69.77	82.78	84.29	88.89	76.75	87.71	79.42	80.97	77.5	84.11	81.96	74.5	66.64	64.75

CD8+ T cell Viability

	Non-Treated						*P. alata*						Isoorientin					
Viable	29.53	27.93	26.03	19.89	19.17	25.17	6.91	5.08	7.93	7.46	3.42	2.69	8.52	4.05	6.25	3.6	7.83	8.4
Early apoptosis	28.45	21.14	27.76	22.3	14.7	12.38	16.51	15.67	13.88	14.18	14.59	13.46	12.47	12.12	14.91	12.75	12.2	
Necrosis/Late apoptosis	42.02	50.93	46.22	57.81	66.14	62.45	76.58	79.25	78.19	78.36	81.98	83.84	79	81.63	81.49	79.42	79.4	

	Vitexin						Catechin						Rutin					
Viable	4.99	5.88	2.05	3.47	4.9	3.98	2.56	5.84	4.31	5.11	3.12	4.63	11.15	15.62	8.13	6.36	10.94	12.23
Early apoptosis	12.11	13.12	10.71	12.04	15.69	14.29	17.95	15.37	19.61	18.62	19.82	16.8	11.68	16.45	13.31	12.97	18.23	12.23
Necrosis/Late apoptosis	82.9	80.99	87.24	78.49	79.41	81.73	79.48	78.79	76.08	76.28	77.06	78.57	77.18	67.9	78.57	80.66	70.83	75.53

DATA FROM FIGURE 5

Lipid Peroxidation - Panel (a)

Positive Control	Non-Treated	*P.alata*
49.12	45.31	64.66
41.37	39.63	60.29
41.35	34.34	41.38
45.63	34.16	40.14
45.28	25.41	36.93
44.25	26.3	35.48
38.13	30.69	63.12
33.81	35.04	34.2
54.34	23.67	34.21
48.15		
33.3		
35.91		
35.03		

Nitric oxide CD4+ T cell - Panel (b)

Positive Control	Non-Treated	*P.alata*
80	11.89	95.27
81.69	15.66	91.63
54.84	15.32	89.12
25	5.94	55.17
25	8.33	42.37
41.67	39.19	62.28
72.73	27.19	74.42
57.69	29.31	71.3
37.5		57.04
40		73.22
26.67		65.78
58.49		78.92
25		76.43

Nitric oxide CD8+ T cell - Panel (c)

Positive Control	Non-Treated	*P.alata*
15.79	4.96	16.19
18.18	2.1	26.13
42.86	4.53	55.48
50	1.87	43.76
57.14	1.92	31.49
21.43	3.61	49.88
20	7.06	50.52
28.57	3.03	45.19
43.75	1.56	52.04

DATA FROM FIGURE 6

Mitochondrial Polarization - Panel (a)

	Depolarized													
Unstimulated	74.97	82.92	83.89	60.9	76.94	69.96	64.85							
Positive control	83.5	83.08	84.03											
Non-Treated	69.68	73.51	80.75	22.5	15.32	60.07	56.98	51.63						
P.alata	97.5	92.79	98.09	84.05	61.11	69.54	62.46							
Isoorientin	58.75	61.56	55.72	63.46	42.93	40.5	50.75	67.89	53.15	59.65	42.7	43.2	61.6	
Vitexin	51.35	54.18	54.09	56.7	58.57	57.52	48.6	45.5						
Catechin	62.35	56.59	57.06	36.5	41.98	29.95	62.48	59.26	68.4	59.53	52.88	58.99	40.74	42.2
Rutin	57.5	70.74	52.68	37.21	31.28	30.82								

	Polarized													
Unstimulated	25.03	17.08	39.1	16.11	23.06	30.04	35.15							
Positive control	16.5	16.92	15.97											
Non-Treated	30.32	26.49	19.25	77.5	84.68	39.93	43.02	48.37						
P.alata	2.5	7.21	1.91	15.95	38.89	30.46	37.54							
Isoorientin	41.25	38.44	44.28	36.54	57.07	59.5	49.25	32.11	46.85	40.35	57.3	56.8	38.4	
Vitexin	48.65	45.82	45.91	43.3	41.43	42.48	51.4	54.5						
Catechin	37.65	43.41	42.94	63.5	58.02	70.05	37.52	40.74	31.6	40.47	47.12	41.01	59.26	57.8
Rutin	42.5	29.26	47.32	62.79	68.72	69.18								

ROS+ CD4+ T cell - Panel (b)

Non-Treated	*P.alata*	Isoorientin	Vitexin	Catechin	Rutin
62.83	69.6	43.28	54.37	50.05	50.2
40.21	73.48	52.91	84.67	38.5	56.8
	94.32	37.48	74.63	48.38	44.48
	80.01	28.09	71.41	34.75	53.97
	86.49	38.43	62.69	33.41	39.91
				52.31	52.05

ROS+ CD8+ T cell - Panel (d)

Non-Treated	*P.alata*	Isoorientin	Vitexin	Catechin	Rutin
37.33	86.56	58.92	36.46	39.68	61.87
42.37	53.7	36.4	45.4	24.89	31.68
	53.29	26.25	57.16	40.02	55.6
	51.7	19.9	58.06	40.34	58.95
	59.56	29.63	66.32	37.28	71.19
	78.98	37.95	58.92	28.3	58.41

mitosox+ CD4+ T cell - Panel (c)

Non-Treated	*P.alata*	Isoorientin	Vitexin	Catechin	Rutin
45.5	59.99	56.36	73.38	59.73	72.26
46.2	62.47	54.56	73.89	55.89	73.88
44.37	69.21	53.98	75.61	58.38	80.05
46.57	65.58	52.64	80.07	54.56	72.19
	68.21	52.97	78.07	57.00	71.79
	67.64	57.1	79.7	57.48	76.63

mitosox+ CD8+ T cell - Panel (e)

Non-Treated	*P.alata*	Isoorientin	Vitexin	Catechin	Rutin
26.34	54.75	62.8	46	56.47	47.42
51.08	42.65	65.7	46.32	55.35	41.56
49.76	41.84	62.64	46.65	57.01	50
48.09	41.54	64.62	41.82	55.79	50
	43.28	52.64	45.91	52.36	50
	48.16	47.86	42.43	51.44	50
